# Optimization of HPLC method for metanephrine and normetanephrine detection in urine: Enhancing diagnostic precision for pheochromocytoma

**DOI:** 10.1016/j.toxrep.2025.101903

**Published:** 2025-01-08

**Authors:** Hafiza Monaza Batool, Madeeha Batool

**Affiliations:** School of Chemistry, University of the Punjab, Lahore 54590, Pakistan

**Keywords:** High-Performance Liquid Chromatography, Metanephrine, Normetanephrine, Pheochromocytoma

## Abstract

Catecholamines and their metabolites play critical physiological roles in the human body. Paragangliomas and pheochromocytomas are rare adrenal tumors that significantly alter catecholamine metabolism, particularly the concentrations of metanephrine (MN) and normetanephrine (NMN). This study presents the development and validation of a rapid and straightforward analytical method using reverse-phase high-performance liquid chromatography (RP-HPLC) coupled with a photodiode array (PDA) detector for quantifying MN and NMN in 24-h urine samples. Sample preparation involved adding 1 mL of urine to a tube containing the internal standard 3-methoxy-4-hydroxy benzylamine hydrochloride (MHBA) and a 2 g/L solution of 2-aminoethyl-diphenylborinate. After vortex mixing and centrifugation, ethyl acetate was used for extraction, and the organic layer was dried under nitrogen at 50–60 °C before reconstitution in the mobile phase. Chromatographic separation was achieved on an RP C-18 column with an isocratic flow of the mobile phase (sodium dihydrogen phosphate, citric acid monohydrate, acetonitrile, and sodium octyl sulfate). Detection was performed at 347 nm, with peak identification based on standard retention times. The method was validated for linearity (10–2000 ng/mL), recovery, sensitivity, precision, accuracy, selectivity, carryover, stability, and dilution effects. It showed a strong correlation coefficient (>0.99) and accuracy within ± 15 %. Inter- and intra-day precision confirmed the method reliability. This validated technique is suitable for clinical and research applications involving catecholamine metabolite screening.

## Highlights

• An RP-HPLC-PDA method for metanephrines was developed, offering a linear range of 10–2000 ng/mL.
• A lower limit of quantification (LLOQ) of 10 ng/mL was achieved for metanephrines.
• A liquid-liquid extraction method was optimized, ensuring high recovery and reproducibility.
• Run time was reduced to 9 min compared to previous techniques.
• The method was validated as sensitive, precise, and superior for clinical metanephrines analysis.

## Graphical Abstract

**Figure:**
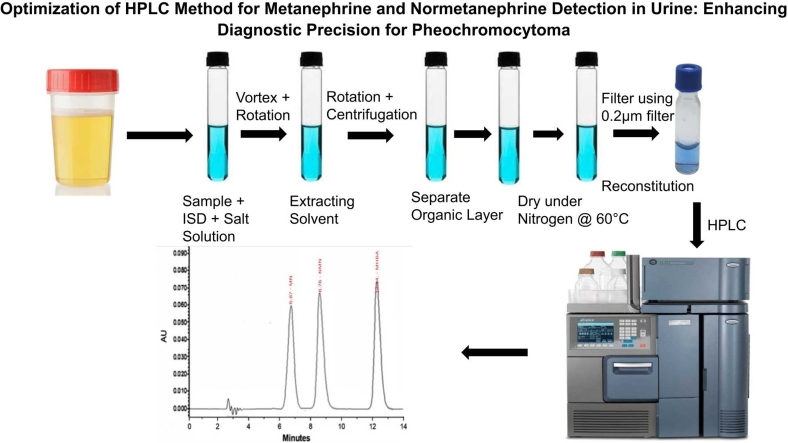

## 1 Introduction

**Fig. 1:**
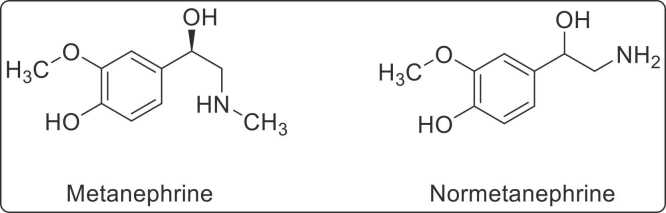
Chemical structures of metanephrine and normetanephrine.

Pheochromocytoma and paraganglioma are rare neuroendocrine tumors arising from the adrenal medulla and sympathetic ganglia, respectively, with most cases occurring in the adrenal glands [1]. These tumors release catecholamines, including dopamine, epinephrine, and norepinephrine, which trigger sympathetic and parasympathetic responses and influence the body's reaction to stress [2], [3]. Although the term pheochromocytoma is often used to refer to both adrenal and extra-adrenal tumors, distinguishing between the two is critical due to differences in malignancy risk and diagnostic approaches [2]. Catecholamine metabolites, such as normetanephrine (NMN) and metanephrine (MN), are essential markers for diagnosing these tumors, as they exhibit more stable concentrations compared to catecholamines, which are secreted intermittently [4]. These metabolites, formed via O-methylation catalyzed by catechol-O-methyltransferase (COMT), undergo further processing in the body. Free metanephrines are converted to vanillylmandelic acid (VMA) by monoamine oxidase (MAO) and are excreted, while conjugated forms are produced in the gastrointestinal tract and also excreted via the kidneys [4], [5]. The diagnostic utility of metanephrines is underscored by their superior sensitivity and specificity compared to catecholamines. Metanephrines are measurable in both plasma (free form) and urine (free and conjugated forms), making them reliable biomarkers for pheochromocytoma and paraganglioma [6]. These tumors, though uncommon, have an annual prevalence of approximately 2–8 cases per 1 million globally, with an incidence of 0.08 per 1 million in Pakistan. However, these figures likely underestimate the true prevalence, as nearly half of the cases are identified only postmortem [1], [7]. Pheochromocytomas primarily occur in individuals aged 40–50 years, with no significant gender bias [3], [8]. **(**Fig. 1**)**

According to the 2014 Endocrine Society Clinical Practice Guideline, supported by level A evidence in the GRADE system, the recommended initial biochemical testing for pheochromocytoma includes measuring plasma-free or urinary fractionated metanephrines (MNs), with no preference for one over the other [9]. Advances in metabolite measurement now focus on measuring free metanephrines in plasma or urine with high sensitivity and minimal interference, enhancing the accuracy of Pheochromocytoma diagnosis [4]. Both plasma and urine are utilized for sample collection, with urine recommended as an alternative specimen due to the challenges in collecting plasma samples and physiological constraints that limit blood collection in the supine position [8], [10]. Enzyme-linked Immunoassays (EIAs) are commonly used analytical methods for metanephrine determination. While these commercially available EIAs are cost-effective and easy to handle, they are associated with a diagnostic sensitivity of only 74.1 %, indicating room for improvement [1]. Various techniques, such as colorimetric assays, immunoassays, gas chromatography-mass spectrometry (GC-MS) and HPLC, have been documented for the measurement of urinary MN and NMN [11]. Advances in the instrumentation of clinical chemical laboratories have led to a transition from traditional fluorometric and spectrophotometric techniques to more modern methods such as high-performance liquid chromatography (HPLC) and immunoassay. These advancements have notably enhanced both the analytical sensitivity and specificity in the field [12]. This study describes an HPLC (high-performance liquid chromatographic) approach designed for the assessment of free and conjugated NMN and MN in urine. The utilization of RP-HPLC with a Photo Diode Array (PDA) detector offers a blend of elevated sensitivity, specificity, and expeditiousness, making it well-suited for routine applications.

## 2 Materials and methods

### 2.1 Standards and chemicals

Urinary based Catecholamine Mix 2 (MN and NMN) standard (1 mL vial, Product number: C-110) having concentration 1 mg/mL Sigma-Aldrich (Cerilliant, Supelco Inc.) and 3-Methoxy-4-hydroxy benzylamine hydrochloride (MHBA) (CBNumber: CB81164697); having concentration 1 mg/mL were purchased from Chemical Books. Blank urine (MN and NMN free urine) (50 mL vial, Product number: SAE0074) was purchased from Sigma-Aldrich. NANO-pure water system (Millipore apparatus, Merck, Germany) was used for water that has been deionized. Ethyl acetate, acetonitrile (HPLC grade) and methanol, NH_3_ solution, 2-aminoethyl-diphenylborinate, sodium dihydrogen phosphate, citric acid monohydrate, and sodium octyl sulfate (analytical grade) bought from Sigma Aldrich.

### 2.2 Sample collection

24-h urine samples were obtained and preserved at 20 °C with a small amount of boric acid as a preservative. Boric acid was used due to its ability to lower the pH of urine, thereby inhibiting bacterial growth and enzymatic activity, preventing the degradation of metabolites, and ensuring the integrity and stability of the samples for accurate analysis up to one month. [13]

### 2.3 Solutions, internal standards, and controls

90:10 v/v solution of water: methanol was prepared by adding 90 mL water and 10 mL methanol in a volumetric flask of 100 mL. 0.25 % NH_4_OH solution in volumetric flask of 100 mL was prepared by adding 780.125 µL of NH_3_ solution. 2 g/L solution of 2-aminoethyl-diphenylborinate was prepared by adding 0.2 g of 2-aminoethyl-diphenylborinate in 100 mL volumetric flask along with 780.125 µL of NH_3_ Solution and making up the volume with 90:10 v/v solution of water: methanol. The mobile phase (0.024 M citric acid monohydrate, 0.1 M sodium dihydrogen phosphate, 9 % acetonitrile and 0.5 mM sodium octyl sulfate, v/v), pH 2.9, was vacuum-filtered using a 0.2 μm hydrophilic polypropylene filter, degassed in an ultrasonic bath prior to use, and kept for a month at 25 °C.

### 2.4 Preparation of calibrators

Internal standards (IS) of 10 μg/mL and 1 μg/mL (1000 ng/mL) were prepared by adding 100 μL of 1.0 mg/mL and 1 mL of the 10 μg/mL from a stock solution of 3-Methoxy-4-hydroxy benzylamine hydrochloride (MHBA) respectively in 10 mL flask and qs the volume with methanol. Stock standard solutions A (10 μg/mL) and B (1 μg/mL) were prepared by adding 100 μL of 1.0 mg/mL and 1000 μL of the 10 μg/mL of standard metabolites (( ± )- (MN) and ( ± )- (NMN)) respectively in a 10-milliliter flask and was diluted with methanol. For the calibration curve (10 μg/mL), and (1 μg/mL) of metabolite solution were transferred into test tubes with screw caps measuring 16 × 125 mm. 100 μL of the 1 μg/mL (1000 ng/mL) of internal standard (IS) (MHBA) were added in all tubes. **(**Table 1**)** To eradicate the solvent effect, all calibrators were dried out by using nitrogen before adding 1 mL of blank urine.

**Table 1:** Illustrated the final concentration of the solution after the addition of blank urine.

**Stock solution (A) of 10 μg/mL (μL)**	**Stock solution (B) of 1 μg/mL (μL)**	**Final concentration of Metanephrine (MN) and Normetanephrine (NMN) (ng/mL)**
200		2000
100		1000
50		500
	200	200
	100	100
	50	50
	10	10

### 2.5 Sample extraction

**Fig. 2:**
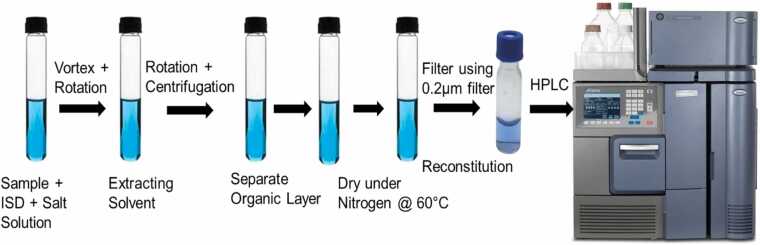
Schematic diagram illustrating the sample preparation for HPLC analysis.

For method assessment, de-identified residual aliquots of 24 h urine were used by evaluating the distribution of the metabolites (MN, NMN). The urine sample was preserved at 20°C with a small amount of boric acid as a preservative. Calibrators and case samples were taken into 16 × 125 mm tubes. 1.0 mL specimens/blank urine, 100 μL of the 1 μg/mL (1000 ng/mL) of internal standard (IS) (MHBA), and 1.5 mL of 2 g/L solution of 2-aminoethyl-diphenylborinate were added in labeled tubes and vortex for a period of 30–60 s. In it, extracting solvent (ethyl acetate), 5 mL was added, rotated at 30 RPM, and then centrifuged for 15 min at 3500 RPM. The topmost layer of organic material was separated and evaporated under nitrogen at 50–60°C. Samples were reconstituted in 200 μL of mobile phase (0.024 M citric acid monohydrate, 0.1 M sodium dihydrogen phosphate, 9 % acetonitrile and 0.5 mM sodium octyl sulfate, v/v) and run on HPLC for analysis. **(**Fig. 2**)**

### 2.6 Instrumentation

#### 2.6.1 Chromatographic conditions

The analytical procedure employed a Waters Alliance e2695 High-Performance Liquid Chromatography (HPLC) Separations Module fitted with a 2998 Photodiode Array (PDA) detector. A 10 µL sample was injected, and chromatographic separation was carried out using the RP C-18 column (5 µm, 4.6 ×150 mm) with pore size 100 Å, which was kept at a temperature of 30°C. The mobile phase (0.024 M citric acid monohydrate, 0.1 M sodium dihydrogen phosphate, 9 % acetonitrile and 0.5 mM sodium octyl sulfate, v/v), pH 2.9, in an isocratic flow, has a 0.5 mL/min flow rate. The wavelength selected for maximum absorption of standards as which maximum area and height were noted through the PDA detector for the simultaneous detection of MN and NMN at a wavelength of 347 nm and characterized using the retention times obtained from HPLC profiles of the standards.

### 2.7 Method validation

**Fig. 3:**
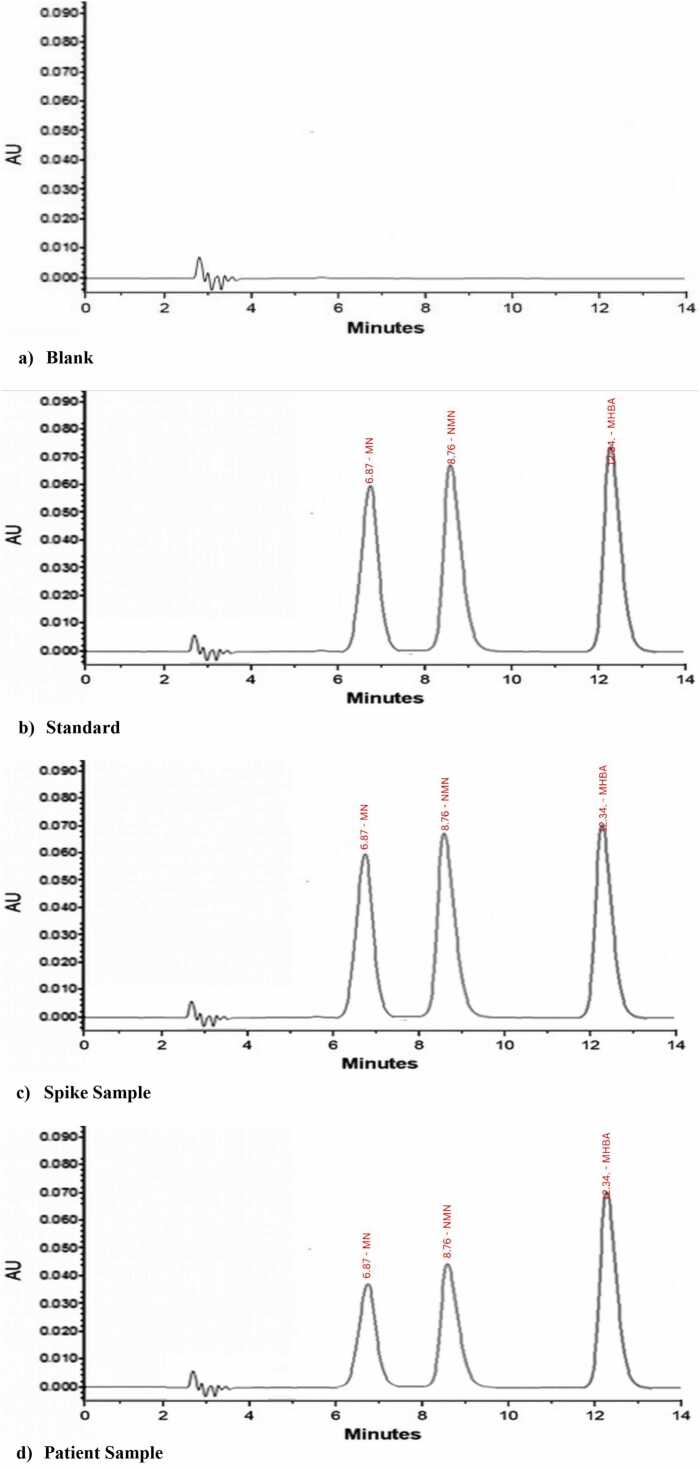
Chromatographic profile using the main solvents in isocratic elution of (a) blank, (b) analyte standard, (c) spiked sample at 2000 ng/mL, and (d) patient sample.

The method was validated in accordance with US Food and Drug Administration (FDA) 2022 recommendations for the industry [14]. The method validation was done by testing linearity (calibration curve) with LLOQ, AMR, recovery, accuracy, precision, stability, selectivity, carryover, and dilution effects. For linearity, blank urine was spiked with varying concentrations of 10, 50, 100, 200, 500, 1000, and 2000 ng/mL of metabolites, followed by a constant concentration (1000 ng/mL) of internal standard. Each level was extracted and run in triplicate. Spiked blank urine samples with different concentrations 10 ng/mL lower limit of quantitation (LLOQ), 100 ng/mL Low(L) (3 times LLOQ), 500 ng/mL Medium (M), and 1000 ng/mL High (H) with five copies were analyzed for inter and intra-day method accuracy on three different days. The acceptable criteria for method validation were accuracy within ± 15 % of the LLOQ ± 20 % of (CV), and the ratio of mass transitions was ± 30 % [15]. The analysis of metabolite-free urine (blank) and standards with the same MN and NMN concentration was spiked for comparison of MN and NMN peak areas. **(**Fig. 3**)**

## 3 Results

### 3.1 Method validation

The chromatograms illustrated in Fig. 3 investigated the method selectivity obtained from the blank (blank urine), analyte standard, spiked samples, and real urine samples.

The blank chromatogram near the analyte's retention period shows no peak and baseline distortion (Fig. 3a). The analytes MN, NMN, and IS (MHBA) showed retention times of 6.87, 8.76, and 12.34 min, respectively. MN and NMN were fully resolved without any interference, producing clear chromatograms in an actual urine sample (Fig. 3b). As for selectivity, blank urine only, free of MN and NMN, was used to rule out the chances of interference with other compounds present in real urine samples (Fig. 3d). In addition to blank urine, we performed spiking experiments by adding known concentrations of MN and NMN into urine samples, allowing us to confirm the resolution of MN and NMN from potential interferences, including structurally similar compounds. The chromatographic conditions were optimized to ensure this specificity, resulting in a clear resolution of the analytes (Fig. 3c).

**Fig. 4:**
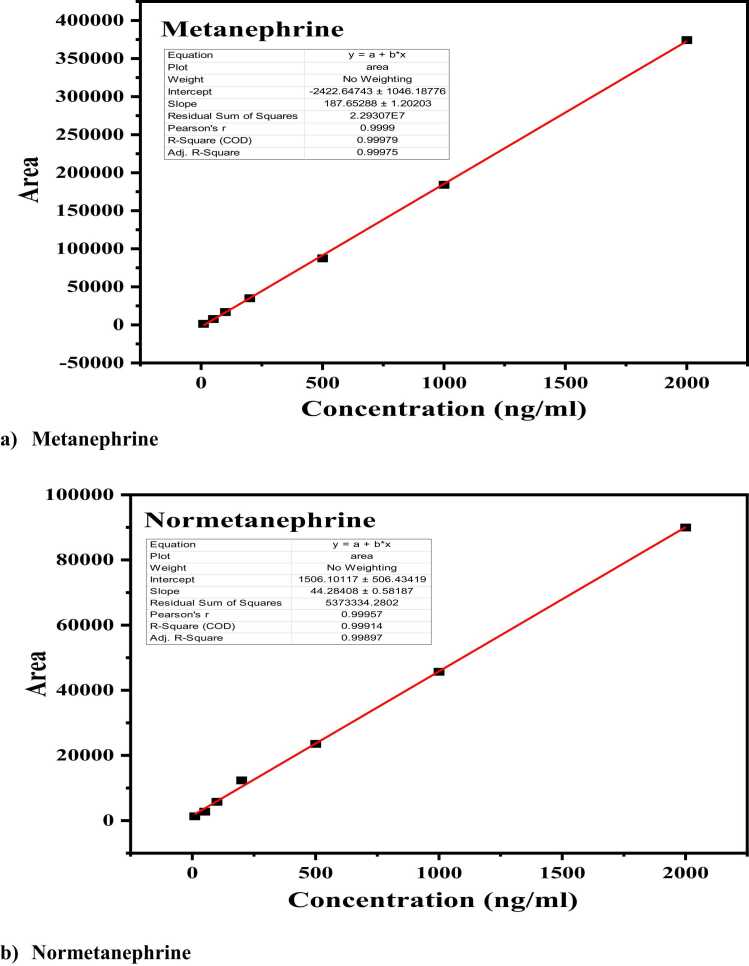
Calibration curves of (a) Metanephrine and (b) Normetanephrine in methanol, using four calibrators with a concentration range of 10–2000 ng/mL.

In Fig. 4, linear regression was performed to assess linearity following FDA guidelines, utilizing seven without a weighting component, calibration points throughout the operational range. The slope and intercept were determined using the equation of the straight line, and the correlation coefficient (r²) was obtained from the regression analysis. Peak areas and analyte concentration were shown to be linearly related within the analytical measurement range (AMR) of 10–2000 ng/mL for both MN and NMN, with determination coefficients (r²) exceeding 0.998. Table 2 provides a summary of the calibration curve parameters for MN and NMN, along with their respective regression coefficients.

**Table 2:** Linear regression data (with no weighing factor) of metanephrine and normetanephrine.

**Sr no.**	**Compound**	**Linearity Range (ng/mL)**	**LLOQ Precision (%CV)**	**LLOQ Accuracy (%Bias)**	**Calibration Parameters**		
					**Intercept**	**Slope**	**r**^**2**^
**1**	Metanephrine	10 – 2000	0.48	−6.56	−2422.64743 ± 1046.18776	187.65288 ± 1.20203	0.9997
**2**	Normetanephrine	10 – 2000	0.12	−6.76	1506.10117 ± 506.43419	44.28408 ± 0.58187	0.9989

The lower limit of quantitation (LLOQ) for MN and NMN was estimated at 10 ng/mL. The method demonstrated sufficient sensitivity to detect MN and NMN in real urine samples at concentrations above 10 ng/mL.

The accuracy and precision of MN and NMN were evaluated at four concentration levels: the lower limit of quantitation (LLOQ), Low (L) at three times the LLOQ, Medium (M), and High (H). For each level, five replicates were run. As shown in Table 3, for MN and NMN, the LLOQ was 10 ng/mL with a bias (accuracy) of 0 – 7 % (<20 %) and precision of < 15 %.

**Table 3:** Accuracy and precision of metanephrine and normetanephrine.

**Sample**	**Nominal value (ng/mL)**	**Accuracy**		**Intra-day and inter-days precision**			
		**Average (ng/mL)**	**Bias (%)**	**Mean (ng/ mL) Intra-day**	**Within day CV (%) n = 10**	**Mean (ng/mL) Inter-day**	**Between Day CV (%) n = 15**
**Metanephrine**							
LLOQ	10	9.34	−6.56	9.30	0.48	9.28	1.36
Low	100	98.69	−1.30	98.60	0.11	98.50	0.13
Mid	500	493.67	−1.26	492.98	0.12	492.58	0.18
High	1000	994.94	−0.50	994.05	0.10	993.81	0.11
**Normetanephrine**							
LLOQ	10	9.32	−6.76	9.31	0.12	9.30	0.14
Low	100	98.34	−1.65	98.33	0.15	98.31	0.02
Mid	500	492.86	−1.42	491.97	0.06	491.59	0.033
High	1000	993.32	−0.66	993.30	0.014	992.7	0.05

All MN and NMN measurements met the accuracy acceptance criteria, as determined by percentage recovery at all seven concentration levels ( ± 15 %). The recovery and matrix effect were assessed by spiking the sample with a known concentration of analytes. The percentage recovery of analytes MN and NMN was found to be 90.2 % for MN and 87.5 % for NMN, respectively, with the matrix effect in an acceptable range. The concentrations-to-evaluate and acceptance criteria were ± 20 %. At every degree of concentration of MN and NMN, the precision was less than 15 % within and between the days. No distortions were observed in the peak or baseline of the blank chromatogram, as shown in Fig. 3. Additionally, there was no interference between the MN and NMN as their chromatograms remained clear even at a lower limit of quantitation (LLOQ). In this case, no carryover was observed, with the response in blank being less than ± 20 % of LLOQ. The stability of the method was analyzed by storing the samples at different conditions for shorter and longer durations. Hence, the approach was suitable for measuring MN and NMN in real urine specimens.

## 4 Discussion

Adrenal tumors, often discovered incidentally during imaging studies, necessitate biochemical and hormonal testing to characterize their nature and guide further treatment [16], [17]. Urinary MN and NMN are widely recognized as critical biomarkers for rare adrenal gland tumors, such as pheochromocytoma and paraganglioma. In this study, we developed and validated an HPLC method for detecting MN and NMN in urinary samples, which demonstrated high specificity, sensitivity, and reproducibility. Application of this method to clinical samples resulted in the detection of clinically significant levels of these metabolites.

Urine samples collected over 24 h were chosen for analysis due to their practical advantages over plasma, including reduced patient distress (eliminating the need for venipuncture) and greater reliability, as urinary concentrations of MN and NMN are less influenced by transient physical or emotional stressors [18], [19].

The extraction protocol, using 2-aminoethyl-diphenylborinate and ethyl acetate, proved to be critical for achieving high specificity and sensitivity. This derivatization agent facilitated stable and reliable detection of catecholamine metabolites, while ethyl acetate was effective in extracting both polar and non-polar compounds. Compared to traditional liquid-liquid extraction (LLE), this approach was simpler, cost-effective, and required less hazardous solvent usage, as supported by findings in the literature [20]. However, the extraction process was time-intensive, highlighting a potential limitation.

Chromatographic separation was optimized using an RP C-18 column (5 µm, 4.6 ×150 mm, 100 Å), maintained at 30°C for the best resolution. The selected wavelength (347 nm) provided optimal peak height and area, ensuring simultaneous detection of MN and NMN. Extensive validation, following FDA guidelines (2022), confirmed the method's linearity, accuracy, precision, selectivity, and stability across multiple conditions. The developed method reliably detected MN and NMN at clinically relevant levels, offering significant utility in diagnosing adrenal tumors.

Over the years, various HPLC methods have been employed for the detection of metanephrine (MN) and normetanephrine (NMN) in urine, showcasing significant advancements in sensitivity, specificity, and runtime. **(**Table 4**)** Early techniques, such as HPLC-FD (1982), achieved detection limits as low as 5 ng/mL but required longer runtimes (12 min) and labor-intensive acid hydrolysis for extraction. [21] Subsequent advancements, including HPLC-ECD methods in the 1990s, incorporated solid-phase extraction techniques, further improving sensitivity and linear range while maintaining runtimes between 12 and 20 min. [22], [23], [24], [25]

**Table 4:** Overview of various reported HPLC methods for the quantification of metanephrine (MN) and normetanephrine (NMN) in urine.

**Year**	**Technique used**	**Extraction technique**	**Linear Assay**	**LLOQ**	**Run Time**	**Reference**
1982	HPLC-FD	Acid Hydrolysis and Ion exchange resins	5–20 ng/mL	5 ng/mL	12 min	[21]
1992	HPLC-ECD	Acid Hydrolysis and Ion exchange resins	MN: upto 20 μM, NMN: upto 70 μM	MN: 0.48 μM, NMN: 1.44 μM	12 min	[22]
1993	HPLC-ECD	Acid Hydrolysis	10–1500 μg/L	NMN: 22 μg/L, MN: 13 μg/L	13 min	[23]
1995	HPLC-ECD	Solid phase Extraction	MN: 5–90 ng/mL, NMN: 5–80 ng/mL	5 ng/mL	15 min	[24]
1997	HPLC-ECD	Solid phase Extraction	5–90 ng/mL,	5 ng/mL	20 min	[25]
2001	HPLC-ECD	Acid Hydrolysis and Ion exchange resins	8–1500 nmol/L	MN: 248 nmol/L, NMN: 434 nmol/L	12 min	[28]
2014	RP-HPLC	Liquid-Liquid Extraction	0.25–15 nmol/mL	0.25 nmol/mL	10 min	[26]
2015	HPLC-CD	Liquid-Liquid Extraction	10–2000 pg/mL	10 pg/mL	10 min	[29]
2016	HPLC-DAD	Solid Phase Extraction	0.06–2 μg/mL	30 ng/mL	9 min	[27]
2021	HPLC-FD and UVD	Solid Phase Extraction	10–2500 μg/L	NMN: 4.6 μg/L, MN: 8.7 μg/L	12 min	[30]
2024	RP-HPLC-PDA	Liquid-Liquid Extraction	10–2000 ng/mL	10 ng/mL	9 min	Present work

More recent developments, such as RP-HPLC and HPLC-DAD methods reported in 2014 and 2016, employed liquid-liquid or solid-phase extraction, achieving detection limits as low as 10 pg/mL with runtimes as short as 9 min. [26], [27] These advancements highlight the continuous evolution of HPLC methodologies, with a focus on enhancing throughput, sensitivity, and robustness for clinical applications.

In comparison, our RP-HPLC-PDA method combines a liquid-liquid extraction technique with a low LLOQ of 10 ng/mL, one of the shortest runtimes (9 min), and a broader linear assay range of 10–2000 ng/mL. These features position our method as a robust and efficient solution for routine clinical analysis, particularly in settings where cost-effectiveness, accessibility, and ease of implementation are critical.

While we acknowledge the possibility of even more sensitive methods emerging, it is essential to consider the socioeconomic context and available laboratory facilities in regions like Pakistan. To our knowledge, this is the first study in Pakistan to develop and validate an HPLC-based method for urinary MN and NMN detection, offering an accessible and reliable diagnostic tool for clinicians managing adrenal tumors.

## 5 Conclusion

The HPLC method developed and validated for the detection of metanephrines (MN) and normetanephrines (NMN) in urine samples demonstrated high sensitivity, selectivity, precision, and accuracy. This approach is well-suited for routine analysis of MN and NMN in patients with suspected adrenal diseases. Additionally, the shorter run times on automated chromatographic systems facilitate the rapid analysis of multiple samples, making the method highly efficient and practical for clinical applications.

## Funding

No funding source to declare.

## CRediT authorship contribution statement

**Madeeha Batool:** Writing – review & editing, Visualization, Supervision, Resources, Project administration, Methodology, Investigation, Conceptualization. **Hafiza Monaza Batool:** Writing – original draft, Visualization, Validation, Software, Methodology, Investigation, Formal analysis, Data curation, Conceptualization.

## Declaration of Competing Interest

The authors declare that they have no known competing financial interests or personal relationships that could have appeared to influence the work reported in this paper.

## Data Availability

No data was used for the research described in the article.
